# Cardiovascular risk factors among high-risk individuals attending the general practice at king Abdulaziz University hospital: a cross-sectional study

**DOI:** 10.1186/s12872-019-1261-6

**Published:** 2019-11-27

**Authors:** Ranya A. Ghamri, Nada S. Alzahrani, Amal M. Alharthi, Hana J. Gadah, Bayan G. Badoghaish, Azzah A. Alzahrani

**Affiliations:** Department of Family Medicine, Faculty of Medicine, King Abdulaziz University, P.O. Box: 42806, Jeddah, 21551 Jeddah, Kingdom of Saudi Arabia

**Keywords:** Cardiovascular, Prevention, Lifestyle, Cardiovascular risk factors, Primary prevention

## Abstract

**Background:**

Cardiovascular disease (CVD) is the primary cause of death worldwide, accounting for 31.0% of all deaths and more than 18 million deaths annually. The 2008 World Health Report indicated that 144 (35%) of the 413 deaths per 100,000 in 2002 in the Kingdom of Saudi Arabia (KSA) were due to CVD. Primary prevention is an important focus of most of the cardiovascular prevention guidelines around the world. In this study, we aimed to describe the prevalence of extrinsic risk factors for CVDs in a high-risk population attending general practice in Jeddah, KSA.

**Methods:**

We conducted a cross-sectional survey at King Abdulaziz University Hospital in Jeddah. Patients started on lipid-lowering and/or antihypertensive and/or antidiabetes treatments without a history of established cardiovascular disease were interviewed. The questionnaire was adopted from the EUROASPIRE III study.

**Results:**

Two hundred and fifty high-risk individuals (80.0% female) were interviewed. Overall, 72% of the patients had been diagnosed with hypertension, 61.2% of patients had dyslipidemia, and approximately two-thirds of patients had diabetes mellitus. Most of the patients (88%) were non-smokers. The mean waist circumference of patients was 101.6 ± 14.1 cm, which suggests most were clinically obese. About 54.8% of the patients followed an unhealthy diet and 52.0% were physically inactive. There were significant differences between women and men in relation to dyslipidemia (*p* = 0.007), unhealthy diet (*p* = 0.034), being overweight (*p* = 0.018), and high blood cholesterol (*p* = 0.002). We observed significantly greater prevalence of hypertension (*p* = 0.073), unhealthy diet (*p* = 0.015), being overweight (p = 0.018), and high blood cholesterol (*p* = 0.000) in those patients with dyslipidemia.

**Conclusion:**

Our study presents novel findings in the KSA. Women were more likely to have high-risk CVD risk factors compared with their male counterparts in this sample. Gender-specific prevention programs in the KSA should be considered to more appropriately target at-risk individuals, to reduce preventable morbidity and mortality associated with CVDs.

## Background

Cardiovascular diseases (CVDs) include coronary heart disease (CHD), cerebrovascular disease, peripheral arterial disease, rheumatic heart disease, congenital heart disease, deep vein thrombosis, and pulmonary embolism [1, 2]. CVD is the primary cause of death worldwide, accounting for 31.0% of all deaths [3] and > 18 million deaths annually [4, 5]. The majority of these deaths occur in low- and middle-income countries [6]. Considerable efforts have been made in high-income countries to define, identify and modify risk factors for CVD in order to develop treatments that promote an age-adjusted decline in CVD mortality [4, 6]. Although a significant proportion of extrinsic risk factors for CVD (such as cigarette smoking, unhealthy diet, and physical inactivity) are preventable, the incidence of CVDs continues to increase because of inadequate prevention measures [7, 8]. Consequently, > 23 million people are projected to die annually from CVD by 2030 [4, 9].

According to the latest World Health Organization (WHO) statistics, chronic, noncommunicable diseases account for the majority of fatalities in the Kingdom of Saudi Arabia (KSA) [10, 11]. The 2008 World Health Report indicated that 144 (35%) of the 413 deaths per 100,000 in 2002 in KSA were due to CVD [10, 11]. CHD represents the third most common cause of hospital-based mortality, behind road traffic accidents and senility [6, 12]. CHD has also been attributed to the increasing and high prevalence of the aforementioned risk factors [6, 13]. An increase in sedentary behavior is likely to be an important driver of CVDs in the KSA [14]. Furthermore, there is a significant proportion of the population with hypertension, a risk factor that contributes significantly to CVD [15].

Studies have shown that primary prevention for people without established CVD is as important as secondary prevention [16, 17]. Indeed, primary prevention is an important focus of most of the cardiovascular prevention guidelines around the world [18–20]. We are not aware of any studies that have focused on CVD risk factors in high-risk individuals in the KSA. Thus, here, we aimed to describe the prevalence of extrinsic risk factors for CVDs in a high-risk population attending general practice in Jeddah, KSA.

## Methods

### Ethical approval

This study was approved by Biomedical Ethics Committee of King Abdulaziz University (Reference No. 322–17). Written consent was obtained from all participants before the interview.

### Study design and area

We conducted a cross-sectional survey at King Abdulaziz University Hospital (KAUH) in Jeddah, the second largest city in Saudi Arabia after the capital city, Riyadh. We selected a random sample of 250 patients attending the General Practice at KAUH. The inclusion criteria were: male and female patients aged > 40 years and currently on antihypertensive and/or lipid-lowering and/or anti-diabetes drug treatment regimens. The exclusion criteria were: patients with established cardiovascular disease (i.e., previous myocardial infarction, unstable angina, stable angina, stroke, peripheral arterial disease, or another atherosclerotic disease).

### Data collection

Data were collected using both anonymous interview-administered questionnaires and physical measurements. The questionnaire was adopted from the EUROASPIRE III study [21] and modified by the researchers. Interviews were conducted by the researchers during hospital working hours. The questionnaire comprised four main sections: (1) personal and demographic details; (2) lifestyle and other risk factors related to smoking, diet, exercise, blood pressure, lipids and glucose; (3) lifestyle changes adopted by the patient over the last 3 years; (4) medications and family history (in patients with premature coronary heart disease). Then, measurements of height, weight, waist circumference, blood pressure, and heart rate were taken. Blood pressure was measured once by a trained nurse on the right or left upper arm in a sitting position using an OMRON automatic sphygmomanometer M5–1. The questionnaire is shown in Additional files 1.

### Statistical analysis

The statistical analysis in this research was performed using SPSS, version 21.0 (IBM, NY, USA). Descriptive statistics such as frequency, percentage, mean, and standard deviation were used to describe the data. A Mann-Whitney test was performed to analyze non-parametric differences in the risk factors for CVD among men and women. A *p*-value < 0.05 was regarded as statistically significant.

## Results

### Demographic and physical characteristics

The demographic characteristics of the 250 patients are shown in Table 1. The mean age of patients was 59.7 ± 11.3 years. The male:female ratio was 1:4 (50:200). Approximately one-third of patients had no formal schooling, while 16.4 and 16.8% of patients had attended primary and secondary school, respectively. Approximately one-third of the patients were unemployed, while one-third spent most of their time at home. Only 31 (12.4%) were still working full-time. The mean height of the patients was 155.3 ± 17.3 cm and the mean weight was 76.4 ± 16.2 kg. The mean waist circumference was 101.6 ± 14.1 cm, which suggests most of the patients were clinically obese. The mean systolic blood pressure level was 139.7 ± 18.1 and the mean diastolic blood pressure level was 71.9 ± 12.1. The mean resting heart rate of the patients was 79.5 ± 14.4 bpm.

**Table 1:** Demographic and physical characteristics of the sample

Characteristics	Number (%) or Mean ± Standard Deviation
Age (years)	59.7 ± 11.3
Gender	
Male	50 (20%)
Female	200 (80%)
Level of Education	
No Formal Schooling	76 (30.8%)
Less than Primary School	12 (4.9%)
Primary School Completed	41 (16.7%)
Secondary School Completed	42 (17.1%)
High School Completed	30 (12.2%)
Intermediate between Secondary Levels	7 (2.8%)
College Completed	30 (12.2%)
Post-Graduate Degree	8 (3.3%)
Job	
Full-Time Employed	31 (12.8%)
Part-Time Employed	5 (2.1%)
Unemployed	85 (35%)
House Person	80 (32.8%)
Retired	42 (17.3%)
Reason for retirement	
Age Related	22 (57.9%)
Other Illness	4 (10.5%)
Personal Choice	12 (31.6%)
Height (cm)	155.3 ± 17.3
Weight (kg)	76.4 ± 16.2
Waist (cm)	101.6 ± 14.1
Body Mass Index (kg/m^2^)	31.24 ± 6.35
Measurement of Systolic BP (mmHg)	139.7 ± 18.1
Measurement of Diastolic BP (mmHg)	71.9 ± 12.1
Measurement of Resting Heart Rate (bpm)	79.5 ± 14.4

### Prevalence of relevant CVD risk factors

As shown in Tables 2, 180 (72%) of the patients had been diagnosed with hypertension with a mean disease duration of 7.9 ± 6.7 years; 153 (61.2%) of patients had dyslipidemia with a mean duration of 6.8 ± 5.8 years. Approximately two-thirds of patients had diabetes mellitus with mean disease duration of 10.75 ± 7.9 years. In this cohort, most of the patients (88%) had never smoked (Table 2). Approximately half the patients had been informed by a healthcare professional that their diet was unhealthy and that they were overweight. One hundred and forty (56%) of the patients claimed that they were actively trying to lose weight, although light physical activity was the most prevalent form of activity (34%). As a limitation to undertaking physical activity, 114 (45.8%) of the patients experienced long-standing illness, disability, or infirmity.

**Table 2:** Relevant CVD Risk Factors

Factors	Number (%) or Mean ***±*** Standard Deviation
History of Hypertension	180 (72%)
Duration of Hypertension (Years)	7.87 ± 6.68
History of Dyslipidemia	153 (61.2%)
Duration of Dyslipidemia (Years)	6.84 ± 5.83
History of Diabetes	172 (68.8%)
Duration of Diabetes (Years)	10.75 ± 7.90
Smoking	
Smoker	17 (6.8%)
Non-smoker	220 (88%)
Former smoker	13 (5.2%)
Diet/Body Weight Issues	
Informed about Unhealthy diet	137 (54.8%)
Informed about being Overweight	133 (53.2%)
Trying to Lose Weight	140 (56%)
Long-Standing Illness	114 (45.8%)
Activity Level	
No Weekly Physical Activity	130 (52%)
Only Light Physical Activity in Most Weeks	85 (34%)
Vigorous Physical Activity Once or Twice a Week	21 (8.4%)
Vigorous Physical Activity Three or More Times a Week	11 (4.4%)
Don’t Know/ Unsure	3 (1.2%)
Blood Pressure	
Informed about High Blood Pressure	185 (74%)
Taking Medicine Prescribed to Lower Blood Pressure	181 (86.6%)
Following a Special Diet to Lower Blood Pressure	90 (43.1%)
Self-Monitoring of Blood Pressure	88 (45.1%)
Cholesterol	
Informed about High Blood Cholesterol	179 (71.6%)
Following a Special Diet to Lower Blood Cholesterol	111 (52.4%)
Diabetes	
Informed about Diabetes	172 (69.9%)
Diabetes	
Treatment	
Diet	2 (1.1%)
Insulin	27 (15.5%)
Oral anti-diabetic drugs	145 (83.3%)
Self-Monitoring of Blood Glucose	113 (63.8%)

Most of the patients (185, 74%) had been informed by a healthcare professional that they had high blood pressure and 181 were taking drugs specifically prescribed to lower their blood pressure. Ninety (43.1%) patients were following a special diet prescribed by a healthcare professional to lower their blood pressure level. One hundred and seventy-nine (71.6%) of the patients had been informed by a healthcare professional that they had high blood cholesterol and 111 (52.4%) were following a special diet prescribed by a healthcare professional to lower their blood cholesterol level. One hundred and seventy-two (69.9%) of the patients had been informed by a healthcare professional that they had diabetes mellitus. The treatment methods for diabetes varied among patients, with oral anti-diabetic drugs (83.3%) being the most commonly used treatment regimen.

### Perception of future CHD risk

Table 3 shows the perceptions and attitudes of the patients concerning their potential risk of CHD; 92 (37.7%) of the patients were worried that they may develop CHD, 81 (32.9%) were neutral, and 73 (29.7%) were not worried. Around half (49.2%) of the patients believed that their risk of developing CHD in the next 10 years was greater than for a person of the same age and sex, while 105 (43%) were neutral, and 19 (7.8%) did not believe they had an increased likelihood of CHD compared to an age- and gender-matched peer.

**Table 3:** Patient Perceptions of Potential Risks of CHD

Attitude	Number (%) or Mean ***±*** Standard Deviation
Worried about Developing CHD	
Strongly Disagree	44 (17.9%)
Disagree	29 (11.8%)
Neutral	81 (32.9%)
Agree	40 (16.3%)
Strongly Agree	52 (21.1%)
Risk Perception of CHD in the Next 10 Years	
Much Higher	82 (33.6%)
Higher	38 (15.6%)
About the Same	105 (43%)
Lower	12 (4.9%)
Much Lower	7 (2.9%)

### Family history of CVD

Family history of CVD was investigated and is reported in Table 4; 27 (10.8%) of the patients in this cohort had a family history of CVD, which was mostly present in brothers (37.1%) and mothers (31.4%). The mean age of being diagnosed with a CVD in those relatives was 47.3 ± 16.8 years. Eleven of the patients’ relatives died due to a CVD; the mean age of death from CVD in those relatives was 66.4 ± 9.1 years.

**Table 4:** Patient Family Histories with Risk Factors for CVDs

Family History	Number (%) or Mean ***±*** Standard Deviation
History of CVD	27 (10.8%)
Relationship	
Father	7 (20%)
Mother	11 (31.4%)
Brother	13 (37.1%)
Sister	4 (11.4%)
Age at Diagnosis of CVD	47.3 ± 16.8
Died of CVD	11 (50%)
Age at Death from CVD	66.4 ± 9.1

### Comparison of CVD risk factors by gender

The differences in CVD risk factors between men and women are shown in Table 5. There were significant differences between men and women in relation to dyslipidemia (*p* = 0.007), unhealthy diet (*p* = 0.034), being overweight (*p* = 0.018), and high blood cholesterol (*p* = 0.002); women were more likely to have the aforementioned CVD risk factors compared with their male counterparts in this sample.

**Table 5:** Prevalence of CVD Risk Factors Between Genders

CVD Risk Factor	Men	Women	P-value
Hypertension	36 (72%)	144 (72%)	0.969
Dyslipidemia	22 (44%)	131 (65.5%)	**0.007**
Diabetes	38 (76%)	134 (67%)	0.250
Smoking	7 (14%)	10 (5%)	0.432
Unhealthy Diet	21 (42%)	116 (58%)	**0.034**
Overweight	20 (40%)	113 (56.5%)	**0.018**
Physical Inactivity	43 (86%)	172 (86%)	0.970
High Blood Cholesterol	27 (54%)	152 (76%)	**0.002**

### Differences in CVD risk factors in patients with hypertension, dyslipidemia and diabetes

We explored whether there were differences in CVD risk factors among patients with hypertension, dyslipidemia, and diabetes (Table 6). We observed significantly greater prevalence of hypertension (*p* = 0.073), unhealthy diet (*p* = 0.015), being overweight (p = 0.018), and high blood cholesterol (*p* = 0.000) in those patients with dyslipidemia. Furthermore, we also determined that those patients without diabetes were significantly more likely to have been diagnosed with hypertension (*p* = 0.001) compared with diabetic patients.

**Table 6:** Differences in CVD Risk Factors among Patients with Hypertension, Dyslipidemia, and Diabetes

CVD Risk Factor	Patients with Hypertension			Patients with Dyslipidemia			Patients with Diabetes		
	Yes	No	*P*-value	Yes	No	P-value	Yes	No	*P*-value
Hypertension	–	–	–	117 (76.5%)	62 (66.0%)	0.073	114 (66.7%)	66 (86.8%)	**0.001**
Dyslipidemia	117 (65.4%)	36 (52.9%)	0.481	–	–	–	108 (63.5%)	45 (59.2%)	0.519
Diabetes	114 (63.3%)	57 (85.1%)	0.815	108 (70.6%)	62 (66.7%)	0.519	–	–	–
Smoking	12 (6.7%)	5 (7.4%)	0.434	8 (5.2%)	9 (9.5%)	0.351	12 (7%)	4 (5.3%)	0.669
Unhealthy Diet	97 (53.9%)	39 (57.4%)	0.899	93 (60.8%)	43 (45.3%)	**0.015**	95 (55.2%)	40 (52.6%)	0.860
Overweight	102 (56.7%)	31 (45.6%)	0.152	90 (58.8%)	42 (44.2%)	**0.018**	93 (54.1%)	40 (52.6%)	0.961
Physical Inactivity	158 (87.8%)	56 (82.4%)	0.573	135 (88.2%)	78 (82.1%)	0.162	150 (87.2%)	64 (84.2%)	0.553
High Blood Cholesterol	135 (75%)	43 (63.2%)	0.676	150 (98%)	27 (28.4%)	**0.000**	125 (72.7%)	54 (71.1%)	0.749

## Discussion

CVD is the primary cause of death worldwide [3]. In developing countries, the prevalence of deaths related to CVD is expected to increase from 28.9% in 1990 to 36.3% by 2020 [22]. CVD is an increasing health concern, especially in the Middle East and the Gulf Council Countries [23, 24], which includes the KSA [12]. The ACE Saudi Arabia study reported a considerable increase in the prevalence of cardiovascular risk factors [12] and a significant increase in the proportion of patients with poor overall control of modifiable risk factors [25]. The urban population of KSA has increased significantly during recent decades, and it is expected to double in the next few years due to lifestyle changes [26, 27]. Within our study population, there was high mean waste circumference; unhealthy diet and a high-level of inactivity; a significantly higher prevalence of key risk factors in women compared with men; and an association between dyslipidemia and other risk factors. We also found that, although our study subjects were selected because they were at high risk of CVD, many did not perceive themselves to be at elevated risk.

Initial prevention, early detection, and robust health-promotion strategies have all been successful in lowering the global burden of CVD [6, 28]. The WHO has identified nine major contributing risk factors for CVD (tobacco smoking, saturated lipids, self-reported hypertension and diabetes, obesity, diet, physical activity, excessive alcohol consumption, and psychosocial factors such as stress), which, in combination, account for 90.4% of the population-attributable risk of an acute myocardial infarction [29]. The present study of 250 patients attending a General Practice clinic in King Abdul-Aziz University Hospital, Jeddah, KSA, focused on the prevalence of six cardiovascular risk factors. In this sample, we determined a current tobacco smoking prevalence of only 6.8%. The majority of the sample (88%) had never smoked tobacco, which suggests that as a risk factor for development of CVD, smoking is less of an issue in KSA than in other parts of the world [30].

A high prevalence of dyslipidemia (61.2%) and hypertension (72%) was notable in this sample. In contrast, the prevalence of dyslipidemia (71.1%) was found to be higher than that of hypertension (43.9%) in a Nigerian study [22]; the level of obesity was also high. Ahmed et al. reported that almost 50% of all patients in their study in the KSA had three or more modifiable risk factors for CVD (diabetes, hypertension, smoking, dyslipidemia, obesity [BMI ≥30 kg/m^2^], or abdominal obesity) [12]. The main risk factors in that sample were dyslipidemia (68.8%), followed by hypertension (41.8%), and diabetes (25.0%) [12]. Similar results were reported in the study by Alsheikh-Ali et al., in which the prevalence rates of dyslipidemia (70%) and abdominal obesity (68%) were higher than those of hypertension (43%) and diabetes (25%) [31]. The majority of outpatients (92%) had at least one modifiable cardiovascular risk factor, 74% had more than one risk factor, and over half (53%) had three or more risk factors [31]. However, in our study, hypertension and diabetes mellitus were slightly more prevalent risk factors than dyslipidemia, affecting two-thirds of the patients.

In the present study, the mean waist circumference of the sample was 101.6 ± 14.1 cm. This is a significant cause for concern, since such large individuals are at very high risk of developing CVD, especially when they have several other risk factors present. The prevalence of obesity, as defined by waist circumference, was also high (68%) in the study by Alsheikh-Ali et al. [31]. Such findings might indicate patients’ unhealthy nutrition choices or lack of opportunities for exercise. Modifiable extrinsic risk factors for the development of all CVDs are to be found in unhealthy lifestyles, including unhealthy dietary habits, physical inactivity, smoking, and being overweight. These factors result in cardiometabolic risk factors, such as hyperglycemia, dyslipidemia and hypertension [32]. In the current study, 54.8% of the patients followed an unhealthy diet and 52.0% were physically inactive. A recent case-control study within KSA demonstrated that sedentary behavior was increasingly associated with risk of CHD [33]. Notably, however, data in Table 3 indicate that more than half of our study population did not believe that they had an increased propensity to CVD, despite the significant risk factors the study population displayed. This may suggest a need for more focused, intelligent and strong education in the KSA on the CVD-related outcomes of factors such as obesity, hypertension, poor diet, lack of exercise and diabetes.

Several studies have reported gender differences in acute coronary syndrome (ACS) with respect to its presentation, diagnosis, clinical management and outcomes. The results of some studies have found that women have higher mortality rates than men; however, gender was not found to be a factor related to the presentation or mortality of patients with ACS in other studies [3, 34]. Several studies have reported a relationship between ACS and gender among Middle Eastern patients [12, 35]. Studies by Mehio Sibai et al., DeNicola et al. and Alshaikh et al. reported high prevalence of obesity among women in oil-rich countries, with the latter two focusing on the KSA, resulting from increasing intake of calories, animal fat and protein, and limitations on physical activity [10, 36, 37]. Our sample was largely comprised of older women who had significantly higher likelihood of dyslipidemia (*p* = 0.007), unhealthy diet (*p* = 0.034), being overweight (*p* = 0.018), and high blood cholesterol (p = 0.007) compared with men in the sample. The majority of these women did relatively little exercise or were unable to do so. Gender-specific prevention programs in the KSA should be considered to more appropriately target at-risk individuals, in order to reduce preventable morbidity and mortality associated with CVDs. Interventions to reduce patient risk in our sample could include changes in drug treatment (including drug(s) used and dosages), education about lifestyle factors, improved diet, and exercise.

Our study does have several limitations. This was a relatively small, single-center cross-sectional study, which may be susceptible to recall bias. Furthermore, the inclusion criteria may not be broadly representative of those at risk of developing CHD. Finally, we did not collect data on socioeconomic status, which would potentially focus prevention efforts in areas where they are most needed.

## Conclusion

Our study presents novel findings in comparison to previous studies within the KSA. Women were more likely to have CVD risk factors including dyslipidemia, unhealthy diet, being overweight, and high blood cholesterol compared with their male counterparts in this sample. Gender-specific prevention programs in the KSA should be considered to more appropriately target at-risk individuals, in order to reduce preventable morbidity and mortality associated with CVDs.

## Supplementary informations

**Additional file 1.** Description: Questionnaire.

## Data Availability

The datasets used and/or analyzed during the current study are available from the corresponding author on reasonable request.

## Abbreviations

**CHD**: Coronary heart disease
**CVDs**: Cardiovascular diseases
**KAUH**: King Abdulaziz University hospital
**KSA**: Kingdom of Saudi Arabia
**WHO**: World health organization
