# Supplementary material for: Cardiovascular risk factors among high-risk individuals attending the general practice at king Abdulaziz University hospital: a cross-sectional study
Source: BMC Cardiovasc Disord. 2019 Nov 27;19:268. doi: 10.1186/s12872-019-1261-6 (PMC6882018; doi:10.1186/s12872-019-1261-6)
Supplement: Supplementary file 1 — Additional file 1. Description: Questionnaire. [file 12872_2019_1261_MOESM1_ESM.doc]

| Site | High Risk Individual Interview Questionnaire |
| --- | --- |
| Patient ID number |  |

### -------------------

### ENTRY CRITERIA

### Has the patient been prescribed one or more of the following medications? □ 1 Yes □ 2 No

- 1. Antihypertensive drug therapy
  2. Lipid-lowering drug therapy
  3. Diabetes therapy (drug therapy or diet alone)

1. **Is the patient free of CHD or other atherosclerotic disease?**  □ 1 Yes □ 2 No

### -------------------

### Information from Medical Notes

| **R1. Date of birth of HRI:** | / *mm/yyyy* |
| --- | --- |
| **R2. Sex:** | □ 1 Male □ 2 Female |

### Information on Risk Factors

| **History of hypertension:** | □ 1 Yes, duration: _____ *months* | □ 2 No | □ 3 Not recorded |
| --- | --- | --- | --- |
| **History of hyperlipidaemia/dyslipidaemia:** | □ 1 Yes, duration: _____ *months* | □ 2 No | □ 3 Not recorded |
| **History of diabetes:** | □ 1 Yes, duration: _____ *months* | □ 2 No | □ 3 Not recorded |

| **Q1. What is the highest level of education you have completed?** | | □ 1 No formal schooling  □ 2 Less than primary school  □ 3 Primary school completed  □ 4 Secondary school completed  □ 5 High school completed | | □ 6 Intermediate between secondary level (e.g. technical training)  □ 7 College/University completed  □ 8 Post graduate degree  □ 9 Insufficient data (unknown) | |
| --- | --- | --- | --- | --- | --- |
| **Q2a. Are you:** | □ 1 Full time employed  □ 2 Part time employed  □ 3 Self employed | | □ 4 Unemployed  □ 5 House person  □ 6 Full time education | | □ 7 Retired  □ 9 Insufficient data (unknown) |
| **Q2b. If retired:** | □ 1 Age related  □ 2 Heart disease related | | □ 3 Other illness  □ 4 Personal choice | | □ 9 Insufficient data (unknown) |

**Risk Factors:**

**Smoking**

| **Q3. Have you ever smoked?** | □ 1 Yes | □ 2 No |
| --- | --- | --- |
| **Q4. For how many years in total have you smoked?** | ________ *years* | |
| **Q5. Do you smoke now?** | □ 1 Yes | □ 2 No |
| **Q6. If smoking no, how many cigarettes do you smoke per day?** | ________ *Cigarettes No/day* | |
| **SG 1. If not smoking now, did you quit within the last 6 months?** | □ 1 Yes | □ 2 No |
| **SG 2. If not smoking now, did you quit more than 6 months ago?** | □ 1 Yes | □ 2 No |
| **SG 3. If not smoking now, at what age did you stop?** | ________ *years* | |
| **SG 4. If smoking now, in the last year how many times have you quit smoking for at last 24 hours?** | ________ *times* | |
| **SG 5. If smoking now, are you seriously thinking of quitting smoking?** | □ 1 Yes, within the next 30 days  □ 2 Yes, within the next 6 months  □ 3 No, not thinking of quitting  □ 9 Don’t know/ Unsure | |

### Diet/ Body Weight

| **Q7.**  **Have you ever been told by a health care professional that your diet is unhealthy?** | □ 1 Yes | □ 2 No | □ 3 Don’t know/ Unsure |
| --- | --- | --- | --- |
| **Q8. Have you ever been told by a health care professional that you are overweight?** | □ 1 Yes | □ 2 No | □ 3 Don’t know/ Unsure |
| **Q9. In the past month, have you been actively trying to lose weight?** | □ 1 Yes | □ 2 No | □ 3 Don’t know/ Unsure |

**Physical Activity**

| **Q10. Do you have any long-standing illness, disability or infirmity?**  *(By long standing it is mean anything that has troubled you over a period of time, or that is likely to affect you over a period of time.)* | | | | |
| --- | --- | --- | --- | --- |
|  | □ 1 Yes | □ 2 No | | □ 3 Don’t know/ Unsure |
| **Q11. Which of the following four best describes your level of activity outside work?** *(Please continue getting to and from work, sporting activity and other physical effort during your leisure time, like gardening or dancing. Vigorous activity causes shortness of breath, a rapid heart rate, and sweating.)* | | | | |
|  | □ 1 No physical activity weekly  □ 2 Only light physical activity in most weeks  □ 3 Vigorous physical activity at least 20 minutes once or twice a week | | □ 4 Vigorous physical activity for at least 20 minutes three or more times a week  □ 9 Don’t know/ Unsure | |

### Blood Pressure

| **Q12. Have you ever been told by a doctor (or other health professional) that you have high blood pressure?** | □ 1 Yes | □ 2 No | □ 3 Don’t know/ Unsure |
| --- | --- | --- | --- |
| **Q13. Are you taking (in the last two weeks) drugs SPECIFICALLY PRESCRIBED to lower your blood pressure?** | □ 1 Yes | □ 2 No | □ 3 Don’t know/ Unsure |
| **Q14. Are you on a special diet prescribed by a doctor (or other health professional) to lower your blood pressure level?** | □ 1 Yes | □ 2 No | □ 3 Don’t know/ Unsure |
| **Q15. If hypertensive, do you monitor your blood pressure yourself?** | □ 1 Yes | □ 2 No | □ 3 Don’t know/ Unsure |

### Cholesterol

| **Q16. Have you ever been told by a doctor (or other health professional) that you have high blood cholesterol?** | □ 1 Yes | □ 2 No | □ 3 Don’t know/ Unsure |
| --- | --- | --- | --- |
| **Q17. Are you on a special diet prescribed by a doctor (or other health professional) to lower your blood cholesterol level?** | □ 1 Yes | □ 2 No | □ 3 Don’t know/ Unsure |

### Diabetes

| **Q18. Have you ever been told by a doctor (or other health professional) that you have diabetes?** | □ 1 Yes | □ 2 No | | | □ 3 Don’t know/ Unsure |
| --- | --- | --- | --- | --- | --- |
| **Q19. If YES, how is it currently being treated?** | □ 1 Diet  □ 2 Insulin | |  | □ 3 Oral anti-diabetic drugs  □ 4 Don’t know / Unsure | |
|  | Section completed | | □ |  | |
| **Q20. If diabetic, do you monitor your blood glucose yourself?** | □ 1 Yes | □ 2 No | | | □ 3 Don’t know/ Unsure |

**Lifestyle Changes**

| **Q21. OVER THE LAST 3 YEARS, which one of the following steps did you take to reduce your risk of heart disease?** | | | |
| --- | --- | --- | --- |
| **Stop smoking**  **- abstinence:** | □ 1 Yes | □ 2 No | □ 3 Unsure/ Don’t know |
| **- reduction:** | □ 1 Yes | □ 2 No | □ 3 Unsure/ Don’t know |
| **- smoking cessation clinic:** | □ 1 Yes | □ 2 No | □ 3 Unsure/ Don’t know |
| **- nicotine replacement therapy:** | □ 1 Yes | □ 2 No | □ 3 Unsure/ Don’t know |
| **- Bupropion:** | □ 1 Yes | □ 2 No | □ 3 Unsure/ Don’t know |
| **- other:** | □ 1 Yes | □ 2 No | □ 3 Unsure/ Don’t know |
| **Healthy diet**  **- reduction of salt intake:** | □ 1 Yes | □ 2 No | □ 3 Unsure/ Don’t know |
| **- reduction of fat intake:** | □ 1 Yes | □ 2 No | □ 3 Unsure/ Don’t know |
| **- reduction of calorie intake:** | □ 1 Yes | □ 2 No | □ 3 Unsure/ Don’t know |
| **- eating more fruits and vegetables:** | □ 1 Yes | □ 2 No | □ 3 Unsure/ Don’t know |
| **- eating more fish:** | □ 1 Yes | □ 2 No | □ 3 Unsure/ Don’t know |
| **Lost weight**  **- following dietary recommendations:** | □ 1 Yes | □ 2 No | □ 3 Unsure/ Don’t know |
| **- weight reducing drugs:** | □ 1 Yes | □ 2 No | □ 3 Unsure/ Don’t know |
| **Increase physical activity**  **- following specific exercise advice from a health or exercise professional:** | □ 1 Yes | □ 2 No | □ 3 Unsure/ Don’t know |
| **- trying to do more general everyday physical activities:** | □ 1 Yes | □ 2 No | □ 3 Unsure/ Don’t know |

| **Q22. Risk Perception** | | | |
| --- | --- | --- | --- |
| **Q22a. Are you worried that you may develop heart disease?** | □ 1 Strongly disagree  □ 2 Disagree | □ 3 Neutral  □ 4 Agree | □ 5 Strongly agree |
| **Q22b. Do you think your risk of getting heart disease in the next 10 years is higher, lower, or about the same as a person of the same age and sex as you?** | □ 1 Much higher  □ 2 Higher  □ 3 About the same | □ 4 Lower  □ 5 Much lower |  |

### Medications

| **Q23. Is the patient on any medication?**  □ 1 Yes **Q29. If YES, please specify ..............................................................................................**  □ 2 No  □ 3 Don’t know/ Unsure |
| --- |

### Q24.Family History (in patients with premature CHD)

| **ID** | **Relationship** | | **History of CVD** | **Age at diagnosis of CVD** | **Died of CVD** | **Age at death from CVD** |
| --- | --- | --- | --- | --- | --- | --- |
| **01** | □ 1 Father  □ 2 Mother  □ 3 Brother | □ 4 Sister  □ 5 Son  □ 6 Daughter | □ 1 Yes  □ 2 No  □ 3 Don’t know/ Unsure | ____ *years* | □ 1 Yes  □ 2 No  □ 3 Don’t know/ Unsure | ___ *years* |
| **02** | □ 1 Father  □ 2 Mother  □ 3 Brother | □ 4 Sister  □ 5 Son  □ 6 Daughter | □ 1 Yes  □ 2 No  □ 3 Don’t know/ Unsure | _____ *years* | □ 1 Yes  □ 2 No  □ 3 Don’t know/ Unsure | ___ *years* |
| **03** | □ 1 Father  □ 2 Mother  □ 3 Brother | □ 4 Sister  □ 5 Son  □ 6 Daughter | □ 1 Yes  □ 2 No  □ 3 Don’t know/ Unsure | _____ *years* | □ 1 Yes  □ 2 No  □ 3 Don’t know/ Unsure | ___ *years* |
| **04** | □ 1 Father  □ 2 Mother  □ 3 Brother | □ 4 Sister  □ 5 Son  □ 6 Daughter | □ 1 Yes  □ 2 No  □ 3 Don’t know/ Unsure | _____ *years* | □ 1 Yes  □ 2 No  □ 3 Don’t know/ Unsure | ___ *years* |

Physical Measurements

**Q25. Measure**

| **Height:** ________ *cm* | **Weight:** ________ *kg* |
| --- | --- |
| **Waist:** ________ *cm* |  |
| **Blood Pressure:** | |
| **measurement of systolic BP:**________ *mmHg* | **measurement of diastolic BP:** ________ *mmHg* |
| **measurement of heart rate:** ________ *bpm* |  |
